# The Effects of β-Alanine Supplementation on Muscle pH and the Power-Duration Relationship during High-Intensity Exercise

**DOI:** 10.3389/fphys.2018.00111

**Published:** 2018-02-21

**Authors:** Matthew I. Black, Andrew M. Jones, Paul T. Morgan, Stephen J. Bailey, Jonathan Fulford, Anni Vanhatalo

**Affiliations:** ^1^Sport and Health Sciences, College of Life and Environmental Sciences, University of Exeter, Exeter, United Kingdom; ^2^School of Sport, Exercise and Health Sciences, Loughborough University, Loughborough, United Kingdom; ^3^NIHR Exeter Clinical Research Facility, University of Exeter, Exeter, United Kingdom

**Keywords:** beta-alanine, carnosine, power-duration relationship, muscle pH, critical power

## Abstract

**Purpose:** To investigate the influence of β-alanine (BA) supplementation on muscle carnosine content, muscle pH and the power-duration relationship (i.e., critical power and W′).

**Methods:** In a double-blind, randomized, placebo-controlled study, 20 recreationally-active males (22 ± 3 y, V°O_2peak_ 3.73 ± 0.44 L·min^−1^) ingested either BA (6.4 g/d for 28 d) or placebo (PL) (6.4 g/d) for 28 d. Subjects completed an incremental test and two 3-min all-out tests separated by 1-min on a cycle ergometer pre- and post-supplementation. Muscle pH was assessed using ^31^P-magnetic resonance spectroscopy (MRS) during incremental (INC KEE) and intermittent knee-extension exercise (INT KEE). Muscle carnosine content was determined using ^1^H-MRS.

**Results:** There were no differences in the change in muscle carnosine content from pre- to post-intervention (PL: 1 ± 16% vs. BA: −4 ± 25%) or in muscle pH during INC KEE or INT KEE (*P* > 0.05) between PL and BA, but blood pH (PL: −0.06 ± 0.10 vs. BA: 0.09 ± 0.13) during the incremental test was elevated post-supplementation in the BA group only (*P* < 0.05). The changes from pre- to post-supplementation in critical power (PL: −8 ± 18 W vs. BA: −6 ± 17 W) and W′ (PL: 1.8 ± 3.3 kJ vs. BA: 1.5 ± 1.7 kJ) were not different between groups. No relationships were detected between muscle carnosine content and indices of exercise performance.

**Conclusions:** BA supplementation had no significant effect on muscle carnosine content and no influence on intramuscular pH during incremental or high-intensity intermittent knee-extension exercise. The small increase in blood pH following BA supplementation was not sufficient to significantly alter the power-duration relationship or exercise performance.

## Introduction

The critical power (CP) model of high-intensity exercise performance is defined by two parameters: the CP, representing the highest sustainable rate of oxidative phosphorylation, and the W′, notionally a fixed energy store derived principally from substrate-level phosphorylation (Monod and Scherrer, 1965; Moritani et al., 1981; Poole et al., 1988; Jones et al., 2008, 2010). Metabolic acidosis, which has been implicated in the fatigue process (Fitts, 1994; Chin and Allen, 1998), develops progressively during exercise >CP, with muscle and blood pH attaining consistent and presumably critically low values at task failure (Poole et al., 1988; Vanhatalo et al., 2010). Consistent with the notion that metabolic acidosis contributes to muscular fatigue, improved muscle H^+^ efflux is related to enhanced intense exercise performance (Hostrup and Bangsbo, 2017). Intramuscular H^+^ accumulation has been shown to interfere with the release of Ca^2+^ from the sarcoplasmic reticulum and thus impair excitation-contraction coupling (Knuth et al., 2006; Debold et al., 2008), inhibit glycolysis (Gevers and Dowdle, 1963; Trivedi and Danforth, 1966; Spriet et al., 1987) and slow PCr recovery following exercise (Harris et al., 1976). The ability to attenuate the rate of muscle H^+^ accumulation during exercise and/or enhance its removal from the muscle during recovery may therefore reduce the extent of exercise-induced disruption to excitation-contraction coupling, glycolytic flux, and PCr recovery and permit increased performance during continuous and intermittent high-intensity exercise.

Carnosine (β-alanyl-L-histidine) is a cytoplasmic dipeptide found in high concentrations within skeletal muscle (Harris et al., 2006), which, due to the pKa of its imidazole side-chain (6.83), is a potent buffer for intramuscular H^+^ accumulation during contractions (Bate-Smith, 1938). β-alanine (BA) has been identified as the rate-limiting substrate in carnosine synthesis (Dunnet and Harris, 1999; Harris et al., 2006) and its ingestion has been shown to increase intramuscular carnosine content (Harris et al., 2006; Baguet et al., 2009; Stellingwerff et al., 2012; Stegen et al., 2013, 2014; Bex et al., 2014) which would presumably improve intramuscular buffering capacity. Some investigations have reported improved high-intensity exercise tolerance and performance following BA supplementation (Hobson et al., 2012; Quesnele et al., 2014; Saunders et al., 2017). However, other studies have shown no significant effect of BA supplementation on performance (Sweeney et al., 2010; Saunders et al., 2012a; Ducker et al., 2013; Jagim et al., 2013), despite subjects following an appropriate supplementation strategy (Hobson et al., 2012) and completing a test ostensibly >CP that would be expected to result in a large decline in muscle pH (Bogdanis et al., 1998; Vanhatalo et al., 2016). Furthermore, despite possible ergogenic effects of BA supplementation being attributed to enhanced intramuscular buffering capacity, no study has assessed differences in muscle pH *during exercise* in humans following BA supplementation. ^31^P-MRS affords the opportunity to non-invasively assess muscle pH *during* exercise with a high temporal resolution.

Given that the W′ has been proposed to represent a work capacity comprising the energy available from the “anaerobic” energy pathways (Moritani et al., 1981; Miura et al., 1999, 2000), it would be expected that an increased glycolytic flux during exercise >CP would increase W′. Attenuating the decline in muscle pH may delay the attainment of a “critical” intramuscular milieu which is associated with the complete utilization of the W′ (Vanhatalo et al., 2010), and thus should be reflected by an increased W′. Enhanced muscle buffering may also expedite the restoration of muscular homeostasis following exercise >CP by permitting enhanced PCr recovery and improving glycolytic flux during a subsequent >CP exercise bout. Improved intramuscular buffering capacity may therefore enhance exercise performance >CP through enhanced W′ and improved W′ recovery kinetics (Skiba et al., 2015).

The purpose of this study was to evaluate the physiological and performance effects of 4 weeks of BA supplementation (6.4 g·d^−1^) on muscle carnosine, muscle pH and the power-duration relationship. It was hypothesized that BA supplementation would: (1) increase the muscle carnosine content at rest; (2) increase intramuscular pH and improve performance during high-intensity, intermittent knee-extension exercise; and (3) increase the size of the W′ estimated in a 3-min all-out cycling test and improve all-out and ramp incremental cycling performance.

## Methods

### Subjects

Twenty healthy male subjects (mean ± *SD*: age 22 ± 3 y, height 1.77 ± 0.07 m, mass 79.0 ± 14.4 kg) volunteered to participate in this study. Prior to testing, subjects were informed of the protocol and possible risks of participation and, subsequently, provided written consent to participate. All procedures were approved by the local Research Ethics Committee and conformed to the code of ethics of the Declaration of Helsinki.

### Experimental design

Subjects visited the laboratory on 5 occasions over a 2-week period pre-supplementation, and on 6 occasions over a 2-week period post-supplementation. Prior to supplementation, baseline muscle carnosine content was determined using ^1^H magnetic resonance spectroscopy (^1^H-MRS) which was followed by incremental knee extension exercise (INC KEE) with muscle metabolic changes assessed via ^31^P magnetic resonance spectroscopy (^31^P-MRS). During another visit, subjects performed a ramp incremental cycling test for the determination of the gas exchange threshold (GET) and V°O_2peak_. On a separate visit, subjects were familiarized to a “repeated 3-min all-out” protocol, which involved the performance of two 3-min all-out tests (described in Burnley et al., 2006; Vanhatalo et al., 2007) separated by 1 min of passive recovery. Subjects returned to the laboratory on a separate occasion to complete an experimental repeated 3-min all-out test. On another occasion, subjects performed an intermittent knee extension exercise protocol (INT KEE) with metabolic changes assessed via ^31^P-MRS. The order of tests was randomized except that: familiarization to the repeated 3-min all-out test preceded the experimental test; and, to set the appropriate work rates, the ramp incremental cycling test and INC KEE preceded the repeated 3-min all-out test and INT KEE, respectively.

In a double-blind, placebo-controlled design, subjects were assigned to BA (SR Carnosyn®, Natural Alternative International, San Marcos CA; sustained release 800 mg tablets e.g., Hill et al., 2007; Smith-Ryan et al., 2012) or a cornflower placebo (PL) group (capsule). Groups were matched for physical characteristics (age, body mass, height), baseline carnosine content, and physiological/fitness measures (V°O_2peak_, GET, CP, W′). Subjects consumed 8 tablets/capsules per day of PL or BA, including two tablets/capsules with every meal (breakfast, lunch, dinner) and two tablets/capsules before bed such that the BA dose was 6.4 g·d^−1^. Following 4 weeks of supplementation, each subject reported to the laboratory to begin post-supplementation tests. Subjects were instructed to continue their supplementation regime during the post-supplementation visits, and therefore supplemented their diet for a total of 6 weeks. The post-supplementation visits comprised the same exercise tests and were performed in the same order as the pre-supplementation visits, with an additional carnosine scan following the completion of all visits after ~6 weeks of supplementation. Subjects were instructed to follow their normal dietary and exercise habits throughout the study. Experimental visits were scheduled at the same time of day (±3 h) and subjects were instructed to report to all testing sessions in a rested and well-hydrated state, having avoided strenuous exercise for 24 h and caffeine for 3 h prior to each test.

### Exercise tests

All cycling tests were performed on the same electronically-braked ergometer (Lode Excalibur Sport, Groningen, The Netherlands). The ergometer seat and handlebars were adjusted for comfort, and settings were recorded and replicated for subsequent visits. The ramp incremental protocol consisted of 3 min of unloaded baseline pedaling followed by a ramp increase in power output of 30 W.min^−1^ until task failure. Subjects were instructed to maintain their self-selected cadence (80 rpm, *n* = 18; 85 rpm, *n* = 1; 90 rpm, *n* = 1) for as long as possible. The test was terminated when the pedal rate fell >10 rpm below the chosen cadence for >5 s despite strong verbal encouragement. V°O_2peak_ was determined as the highest 30-s mean value. The GET was established from the gas exchange data averaged in 10-s time bins using the following criteria: (1) the first disproportionate increase in V°CO_2_ vs. V°O_2_; (2) an increase in minute ventilation (V°_E_) relative to V°O_2_ with no increase in V°_E_/V°CO_2_; and (3) the first increase in end-tidal O_2_ tension with no fall in end-tidal CO_2_ tension.

The repeated 3-min all-out test began with 3 min of baseline pedaling (20 W), at the same self-selected cadence chosen during the ramp incremental test, followed by two 3-min all-out efforts separated by 1 min of active recovery (20-W cycling). Subjects were asked to accelerate to 110–120 rpm over the final 5 s of the baseline period, and for the final 5 s of the active recovery. The resistance on the pedals during the 3-min all-out test was set using the linear mode of the ergometer such that, on reaching their preferred cadence, the power output would be equivalent to 50% of the difference between the power output at GET and V°O_2peak_ (linear factor = power/cadence^2^). To ensure an all-out effort, subjects were instructed and strongly encouraged to attain their peak power output as quickly as possible, and to maintain their cadence as high as possible until instructed to stop. CP was estimated as the mean power output during the final 30 s of bout 1. End test power (EP) was determined as the mean 30 s power output during the final 30 s of bout 2. Bout 1 W′ was defined as the amount of work done above bout 1 CP. During bout 2, the amount of work performed above bout 1 CP (W > CP), was determined to provide a measure of W′ recovery. The V°O_2peak_ during each bout of the repeated 3-min all-out test was calculated as the highest 15 s rolling mean value.

To assess muscle metabolism during exercise, subjects performed single-legged knee-extension exercise in a prone position within a magnetic resonance scanner, as described by Vanhatalo et al. (2010). The INC KEE consisted of 30 s of exercise lifting 1 kg, followed by a 0.5 kg increase in mass every 30 s until task failure. For the INT KEE exercise protocol, the weight within the load basket was 120% of the peak work rate recorded in the ramp incremental test with the protocol consisting of 60 s of high-intensity work, followed by 18 s of passive rest, with the sequence repeated until task failure.

### Pulmonary gas exchange

Breath-by-breath pulmonary gas exchange data were collected continuously during all cycling tests, with subjects wearing a nose clip and breathing through a low-dead space, low resistance mouthpiece and impeller turbine assembly (Triple V, Jaeger, Hoechberg, Germany). The inspired and expired gas volume and gas concentration signals were sampled continuously at 100 Hz, the latter using paramagnetic (O_2_) and infrared (CO_2_) analysers (Oxycon Pro, Jaeger) via a capillary line connected to the mouthpiece. These analysers were calibrated before each test with gases of known concentration, and the turbine volume transducer was calibrated using a 3-L syringe (Hans Rudolph, KS). The volume and concentration signals were time-aligned, accounting for the transit delay in capillary gas and analyser rise time relative to the volume signal. The V°O_2_, V°CO_2_ and V°_E_ were calculated for each breath using standard formulae.

### Blood analyses

Venous blood samples were drawn into 5-mL lithium heparin tubes (Terumo Corporation, Leuven, Belgium) at baseline and at discrete time-points during the 3-min all-out test (bout 1 and 2: 30, 60, 90, 170, and 30 s post-test) and the ramp incremental test (120, 240, 360, 480, 600 s, T_lim_, T_lim_ +60 s, T_lim_ +120 s) from a cannula (Insyte-W™, Becton-Dickinson, Madrid, Spain) inserted into the subject's antecubital vein. The blood was analyzed for lactate concentration ([La]) (YSI 2300, Yellow Springs Instruments, Yellow Springs, OH) and 1.5 mL of whole blood was extracted and stored at −80°C for subsequent determination of pH.

### MRS measurements

MRS measurements were performed within the bore of a 1.5 T superconducting magnet (Gyroscan Clinical Intera, Philips, The Netherlands). Muscle carnosine content was measured in the vastus medialis (VM), vastus lateralis (VL), and rectus femoris (RF) muscles using ^1^H-MRS. Subjects were secured to the scanner bed in the supine position via Velcro straps which were fastened across the thigh to minimize movement during the scans. Following the acquisition of a localiser series, a high-resolution coronal imaging series of the thigh was acquired to allow identification of the lateral and medial condyles (Fast spin echo, echo train 19, repetition time of 2,660 ms, echo time of 13 ms, slice 4 mm, pixel 0.625 × 0.625 mm). A location within the center of the thigh was selected from the coronal images relative to the condyles using measurement tools contained within the scanner software. A transverse image was then acquired at this location (fast spin echo repetition time of 115 ms, echo time of 15 ms, slice thickness 4 mm, pixel 0.98 × 0.98 mm) and used for the placement of the MRS voxels (20 [anterior-posterior) × 30 (right-left) × 50 (foot-head) mm for individual muscles, 30 × 70 × 50 mm for voxel centered over the quadriceps). Single-voxel point resolved spectroscopy was undertaken with a 4-element flexible surface coil (45 cm diameter right-left, 30 cm diameter foot-head) with the following parameters: repetition time of 2,000 ms, echo time of 30 ms, 128 excitations, 1,024 data points, spectral bandwidth of 1,200 Hz, and a total acquisition time of 4.24 min. The sequence included a range of preparation phases, including the determination of the water resonance frequency, 90 degree pulse power calibration, shimming and gradient adjustments. For the repeat visits, scout and coronal images were again acquired and the transverse slice position replicated by placing the slice at the same distance from the condyles as for the baseline visit. The integral of the carnosine-H2 peak [at ~8 parts per million (ppm)] was quantified relative to the water peak integral (×1,000) using the AMARES fitting algorithm in the jMRUI (version 3) software package (http://www.mrui.uab.cat/mrui). Muscle [carnosine] was expressed as a ratio relative to the water peak. To determine the reliability of this assessment, a separate cohort of 6 subjects visited the laboratory on consecutive days for the determination of baseline muscle carnosine content. Subjects were instructed to arrive at the laboratory well-hydrated and rested, having avoided strenuous exercise for 24 h prior to the assessment. Each scan was performed at the same time of day (±2 h) for each individual.

Concentrations of phosphorous-containing muscle metabolites and pH during exercise were determined as previously described (Vanhatalo et al., 2010). Briefly, a 6 cm ^31^P transmit/receive surface coil was placed within the ergometer bed, and the subject was positioned so that the coil was centered over the quadriceps muscle of the right leg. Initially, fast field echo images were acquired to determine the correct positioning of the muscle in relation to the coil. A number of pre-acquisition steps were performed to optimize the signal from the muscle under investigation, and tuning and matching of the coil were performed to maximize energy transfer between the coil and the muscle. To ensure that the muscle was consistently at the same distance from the coil at the time of data sampling, the subjects matched their movement (i.e., extension and flexion of the knee) to an audible cue. Data were acquired every 1.5 s with a spectral width of 1,500 Hz and 1,000 data points. Phase cycling with four phase cycles was employed, leading to a spectrum being acquired every 6 s. Intracellular pH was calculated using the chemical shift of the P_i_ spectra relative to the PCr peak (Taylor et al., 1983). The [PCr] and [P_i_] were expressed as percentage change relative to resting baseline, which was assumed to represent 100%. Resting and end-exercise values of [PCr], [P_i_], and pH were calculated over the last 30 s of the rest or exercise period.

### Statistical analyses

Independent samples *t*-tests were used to assess differences between the groups prior to supplementation. Analysis of variance (ANOVA) with repeated measures was used to test for differences in V°O_2peak_ between the ramp incremental cycling test and bout 1 and 2 of the repeated 3-min all-out cycling test. A repeated measures analysis of covariance (ANCOVA), with baseline values used as the covariate, was used to test for differences between supplementation groups in the ramp incremental test (V°O_2peak_, GET, peak power output, blood pH and [La]), the repeated 3-min all-out test (CP, EP, W′, W>CP, peak power output, total work done (TWD), blood pH and [La]), the incremental- and intermittent knee extension exercise tests (muscle pH, [P_i_] and [PCr]), and muscle carnosine content. All significant main and interaction effects were followed up using Fisher's least significant difference post hoc tests. The coefficient of variation (CV %) was used to assess the day-to-day variability in determination of muscle carnosine content. Pearson's product-moment correlation coefficients were used to assess the association between muscle carnosine content and performance. All data are presented as mean ± *SD*. Statistical analysis was performed using SPSS version 22 (SPSS Inc., Chicago, Illinois, USA) with significance set as *P* < 0.05.

## Results

There were no significant differences in physical characteristics or physiological/fitness measures at baseline between the PL and BA supplementation groups (Table 1). No subject reported any adverse effects of supplementation, and self-report supplementation diaries, which were issued weekly, confirmed adherence to the supplementation regimen. Eleven subjects correctly identified the supplementation group to which they were assigned (BA, *n* = 6; PL, *n* = 5).

**Table 1:** Group mean (± *SD*) baseline physical characteristics and physiological responses to the ramp incremental cycling test, bout 1 of the repeated 3-min all-out cycling test, and whole thigh muscle carnosine content for the placebo (PL) and β-alanine (BA) groups.

	**PL**	**BA**
**PHYSICAL CHARACTERISTICS**		
Mass (kg)	81.0 ± 18.1	77.1 ± 10.0
Age (y)	21 ± 3	23 ± 4
**PHYSIOLOGICAL CHARACTERISTICS**		
Ramp test V°O_2peak_ (L·min^−1^)	3.81 ± 0.52	3.64 ± 0.28
Ramp test peak power (W)	350 ± 49	344 ± 30
GET (W)	125 ± 15	115 ± 16
CP (W)	236 ± 47	232 ± 32
W′ (kJ)	17.8 ± 5.6	18.5 ± 1.8
Muscle carnosine (whole thigh) (A.U.)	0.228 ± 0.132	0.203 ± 0.082

*The post-supplementation whole thigh muscle carnosine values are presented as the mean of the two post-supplementation scans at 4 and 6 weeks. No significant differences were observed between the groups (P > 0.05)*.

### Muscle carnosine content

**Figure 1:**
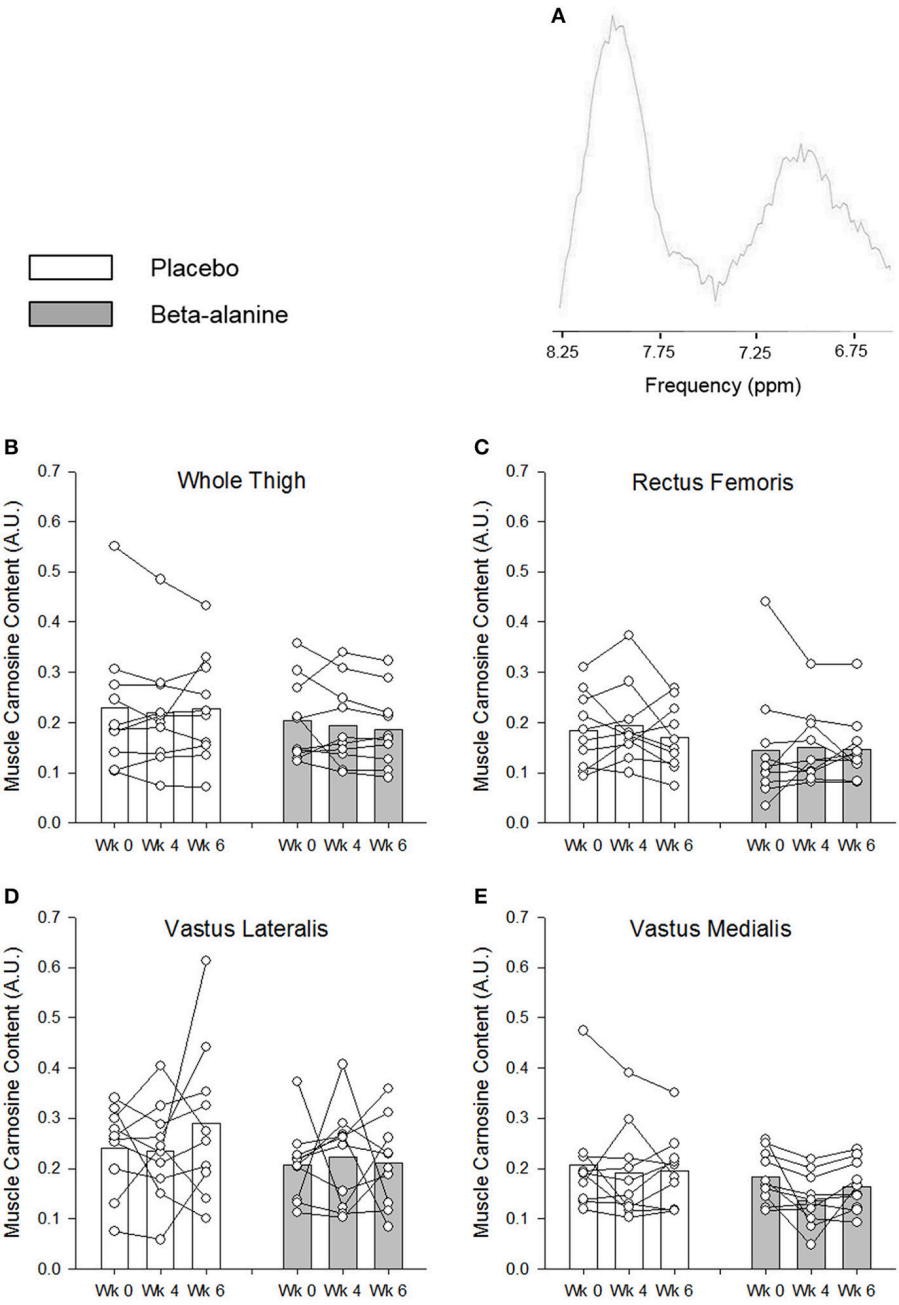
A representative ^1^H-MRS spectrum is provided in **(A)**. The muscle carnosine content for the placebo (white) and β-alanine (gray) groups for the whole quadriceps **(B)**, rectus femoris **(C)**, vastus lateralis **(D)**, and vastus medialis **(E)**. Solid lines indicate individual responses in muscle carnosine content. For clarity, error bars were omitted.

BA supplementation did not significantly increase muscle carnosine content following 4 weeks of supplementation in the VM (Week 0, 0.183 ± 0.051 vs. Weeks 4, 0.137 ± 0.053), VL (Week 0, 0.207 ± 0.074 vs. Weeks 4, 0.223 ± 0.098), RF (Week 0, 0.145 ± 0.116 vs. Weeks 4, 0.151 ± 0.072), or across the whole thigh (Week 0, 0.203 ± 0.082 vs. Weeks 4, 0.194 ± 0.083; Figure 1). Similarly, muscle carnosine content was not significant increased from baseline in the VM (0.164 ± 0.049), VL (0.211 ± 0.086), RF (0.147 ± 0.068), or across the whole thigh (0.186 ± 0.076) following 6 weeks of supplementation (Figure 1). No significant differences in muscle carnosine content were observed following 4 and 6 weeks of supplementation (Figure 1). The day-to-day variability (CV %) in the assessment of baseline muscle carnosine content by ^1^H-MRS was 15 ± 8% (range: 3% to 26%). The variability in the assessment of carnosine in the placebo group between baseline and following 4 weeks of supplementation was 15 ± 13% (range: 0–51%), 18 ± 16% (range: 0–74%) following 6 weeks of supplementation, and 18 ± 16% (range: 0–76%) between weeks 4 and 6.

### Muscle and blood pH

**Figure 2:**
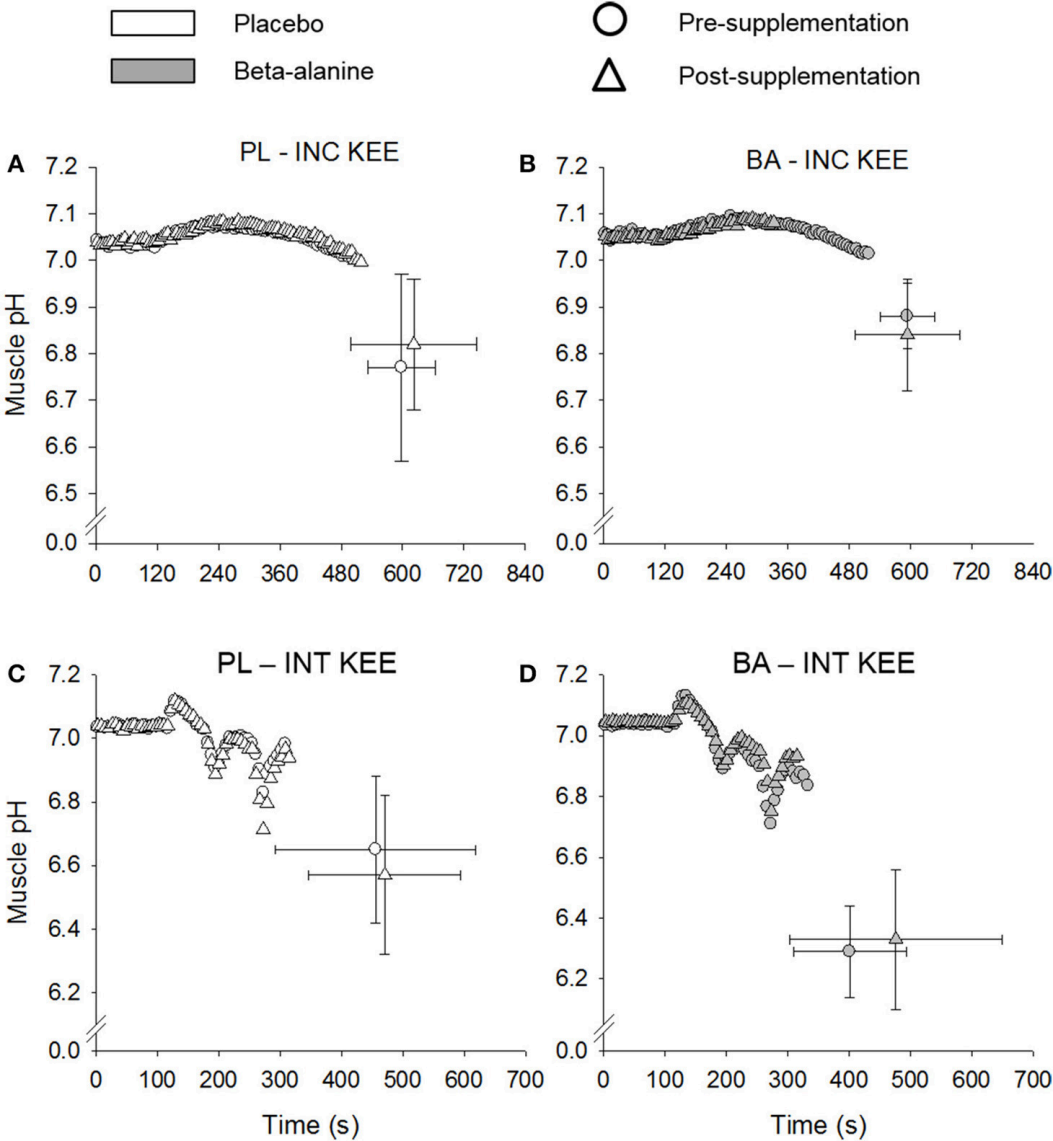
The placebo (PL; white) and β-alanine (BA; gray) group mean muscle pH response during incremental (INC KEE) **(A,B)** and intermittent (INT KEE) **(C,D)** knee-extension exercise pre- (circles) and post- (triangles) supplementation. Error bars represent *SD*. For clarity, error bars are omitted for all data points except T_lim_.

No differences were observed in muscle pH between the supplementation groups (BA vs. PL), at rest, at T_lim_, or at any time-point during INC KEE or INT KEE (Figure 2). No between group changes were observed in muscle [P_i_] and [PCr] during INT KEE and INC KEE (*P* > 0.05) (Table 2).

**Table 2:** Muscle phosphocreatine ([PCr]) and inorganic phosphate ([P_i_]) concentrations and pH at T_lim_ during incremental (INC KEE) and intermittent (INT KEE) knee extension exercise pre- and post-supplementation for the placebo (PL) and β-alanine (BA) groups.

	**INC KEE**				**INT KEE**			
	**PL**		**BA**		**PL**		**BA**	
	**Pre**	**Post**	**Pre**	**Post**	**Pre**	**Post**	**Pre**	**Post**
End-exercise [PCr] (%)	53 ± 13	52 ± 9	52 ± 9	51 ± 10	49 ± 9	47 ± 12	40 ± 8	44 ± 10
End-exercise [P_i_] (%)	442 ± 132	412 ± 73	418 ± 105	431 ± 91	422 ± 84	414 ± 110	510 ± 134	469 ± 58
Baseline pH	7.03 ± 0.02	7.04 ± 0.04	7.05 ± 0.03	7.05 ± 0.01	7.04 ± 0.02	7.04 ± 0.02	7.04 ± 0.01	7.05 ± 0.02
End-exercise pH	6.77 ± 0.20	6.82 ± 0.14	6.88 ± 0.07	6.84 ± 0.12	6.65 ± 0.23	6.57 ± 0.25	6.29 ± 0.15	6.33 ± 0.23
ΔpH	−0.26 ± 0.20	−0.22 ± 0.15	−0.14 ± 0.03	−0.19 ± 0.12	−0.38 ± 0.24	−0.46 ± 0.24	−0.75 ± 0.16	−0.72 ± 0.24

*[PCr] and [Pi] are expressed relative to resting baseline*.

**Figure 3:**
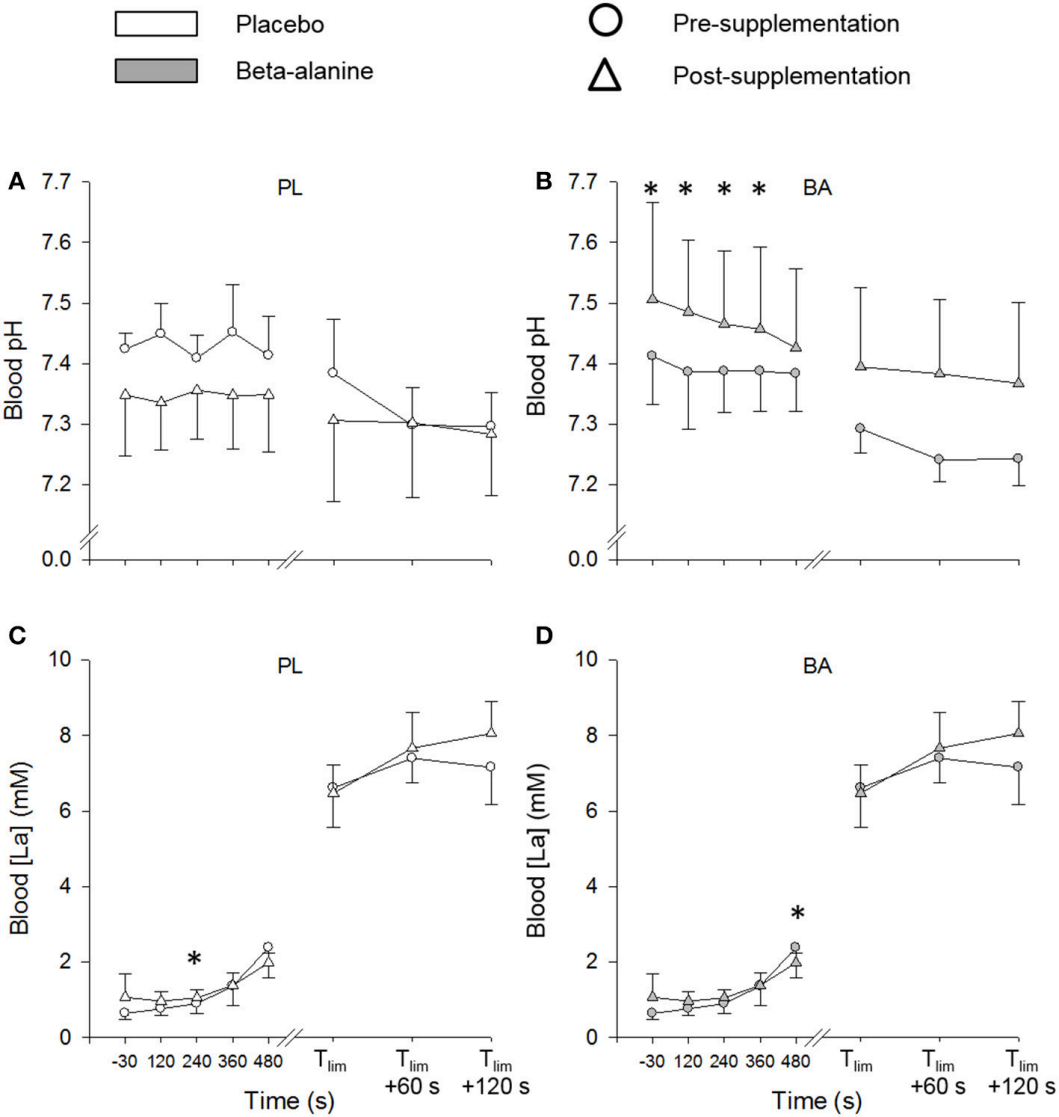
The placebo (white) and β-alanine (gray) group mean blood pH **(A,B)** and blood lactate ([La]) **(C,D)**, during the pre- (circles) and post-supplementation (triangles) ramp incremental test. Significant between group differences are denoted by ^*^P < 0.05. Error bars represent *SD*.

The BA group had a greater blood pH post supplementation compared to the PL group at discrete time-points during ramp incremental cycling (*P* < 0.05) (Figure 3). Blood [La] was significantly greater in the BA group following 480 s of ramp incremental cycling (*P* < 0.05), and remained significantly elevated at exhaustion (*P* < 0.05) (Figure 3). There were no significant between group differences in blood pH or [La] at any time-point during the repeated 3-min all-out test (*P* > 0.05).

### Power-duration relationship

**Figure 4:**
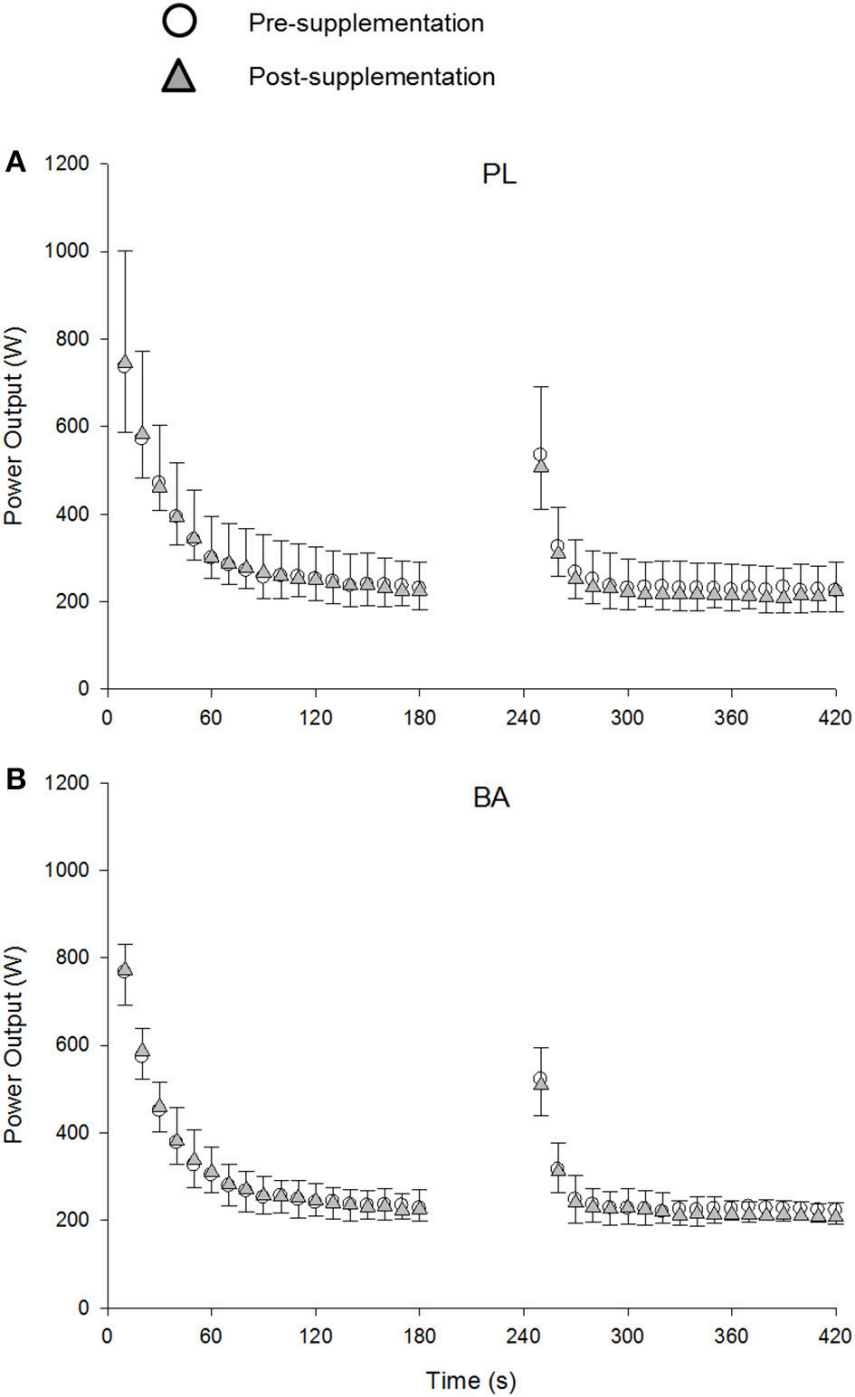
The group mean power profiles during the repeated 3-min all-out test for placebo (PL; **A**) and β-alanine (BA; **B**) groups pre- (circles) and post- (triangles) supplementation. The data are shown every 10s. *SD* is displayed by negative error bars for the pre-, and positive error bars for the post-supplementation trials.

**Figure 5:**
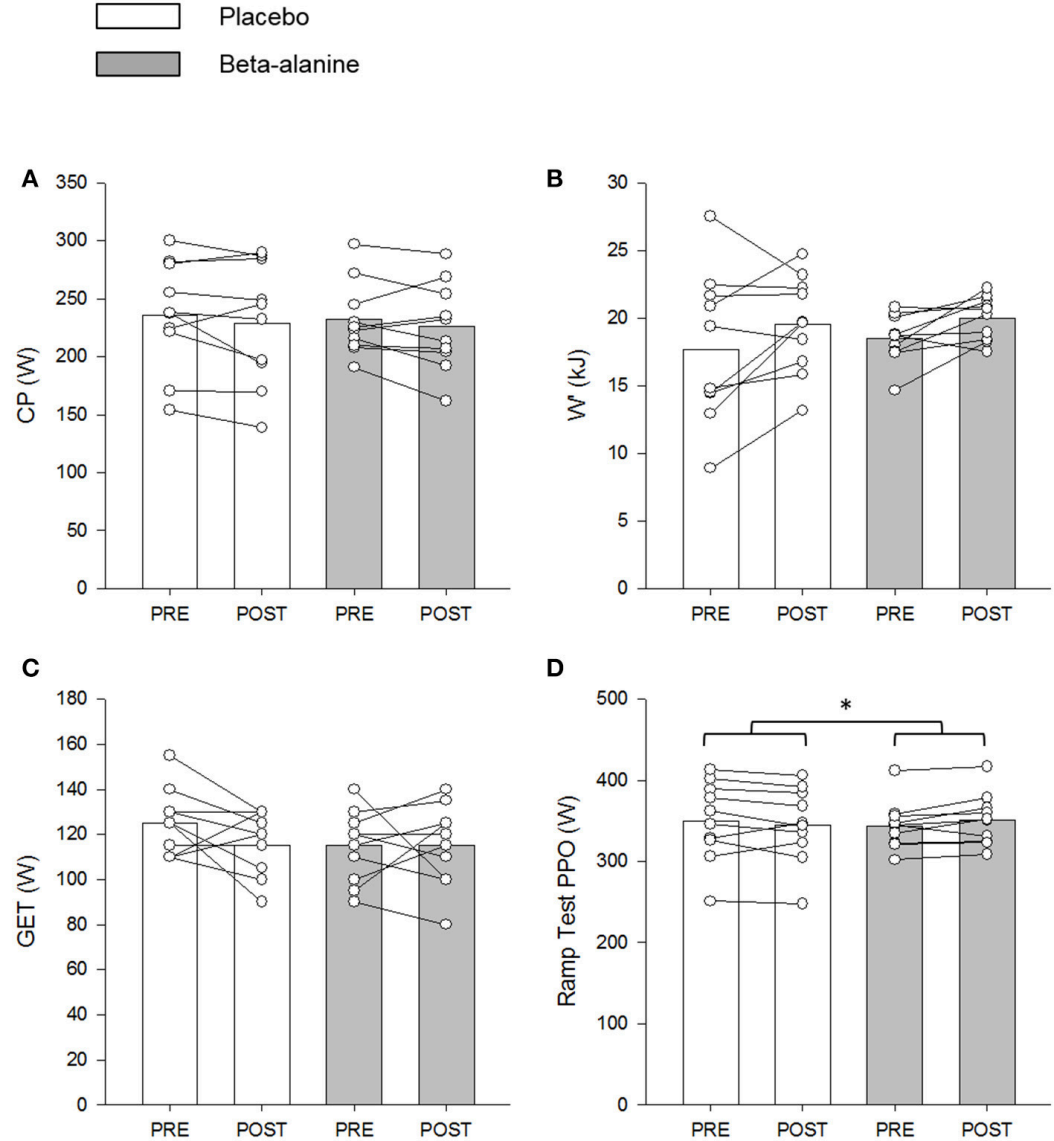
The placebo (PL; white) and β-alanine (BA; gray) group mean and individual critical power (CP; **A**) and W′ **(B)** determined during the 3-min all-out test, and power output at GET **(C)** and peak power output (PPO) **(D)** determined during the ramp incremental test. Pre- and post-supplementation data are presented as circles and triangles, respectively. Significant interaction effects are denoted by ^*^P < 0.05.

The group mean power profiles for both bouts of the repeated 3-min all-out test are displayed in Figure 4. The V°O_2peak_ values attained during bout 1 (Pre: 3.90 ± 0.52 L.min^−1^; Post: 3.75 ± 0.48 L.min^−1^) and bout 2 (Pre: 3.97 ± 0.40 L.min^−1^; Post: 3.79 ± 0.47 L.min^−1^) of the repeated 3-min all test were not significantly different from V°O_2peak_ (Pre: 3.82 ± 0.41 L.min^−1^; Post: 3.68 ± 0.41 L.min^−1^) achieved during the ramp incremental test (*P* > 0.05). There were no significant between group differences for V°O_2peak_, CP or EP, W′ or W > CP, TWD and peak power output following supplementation (*P* > 0.05) (Table 3; Figure 5). No significant correlations were observed between the absolute or percentage change in whole muscle carnosine content or the blood [La] or blood [pH], and the absolute or percentage change in CP, W′, TWD, or peak power output (all *P* > 0.05).

**Table 3:** Group mean (± *SD*) critical power (CP), W′, total work done (TWD), end test power (EP) and work done above CP (W > CP) determined during bout 1 and 2 of the 3-min all-out test pre- and post-supplementation.

		**PL**		**BA**	
		**Pre**	**Post**	**Pre**	**Post**
Bout 1	CP (W)	236 ± 47	229 ± 52	232 ± 32	226 ± 38
	W′ (kJ)	17.8 ± 5.6	19.5 ± 3.6	18.5 ± 1.8	20.0 ± 1.6
	TWD (kJ)	60.3 ± 8.6	60.7 ± 9.1	60.2 ± 6.4	60.6 ± 7.4
	Peak power output (W)	858 ± 184	930 ± 162	960 ± 137	946 ± 100
Bout 2	EP (W)	226 ± 52	210 ± 51	222 ± 25	211 ± 31
	W > CP (kJ)	5.6 ± 2.7	4.0 ± 4.0	5.0 ± 2.4	4.3 ± 3.2
	TWD (kJ)	48.1 ± 9.6	45.2 ± 9.2	46.7 ± 5.3	44.9 ± 6.2
	Peak power output (W)	741 ± 190	732 ± 163	700 ± 74	741 ± 125

*No significant interaction effects (group × time) were observed*.

### Ramp incremental cycling test performance

There was a significant between group difference for peak power output (*P* < 0.05), which was greater in the BA group from pre- to post-supplementation (Δ: 7 ± 10 W), relative to the PL group (Δ: −5 ± 13 W) (Figure 5). No between group differences were observed for the GET estimated in the ramp incremental test (*P* > 0.05) (Figure 5).

### Knee-extension exercise performance

**Figure 6:**
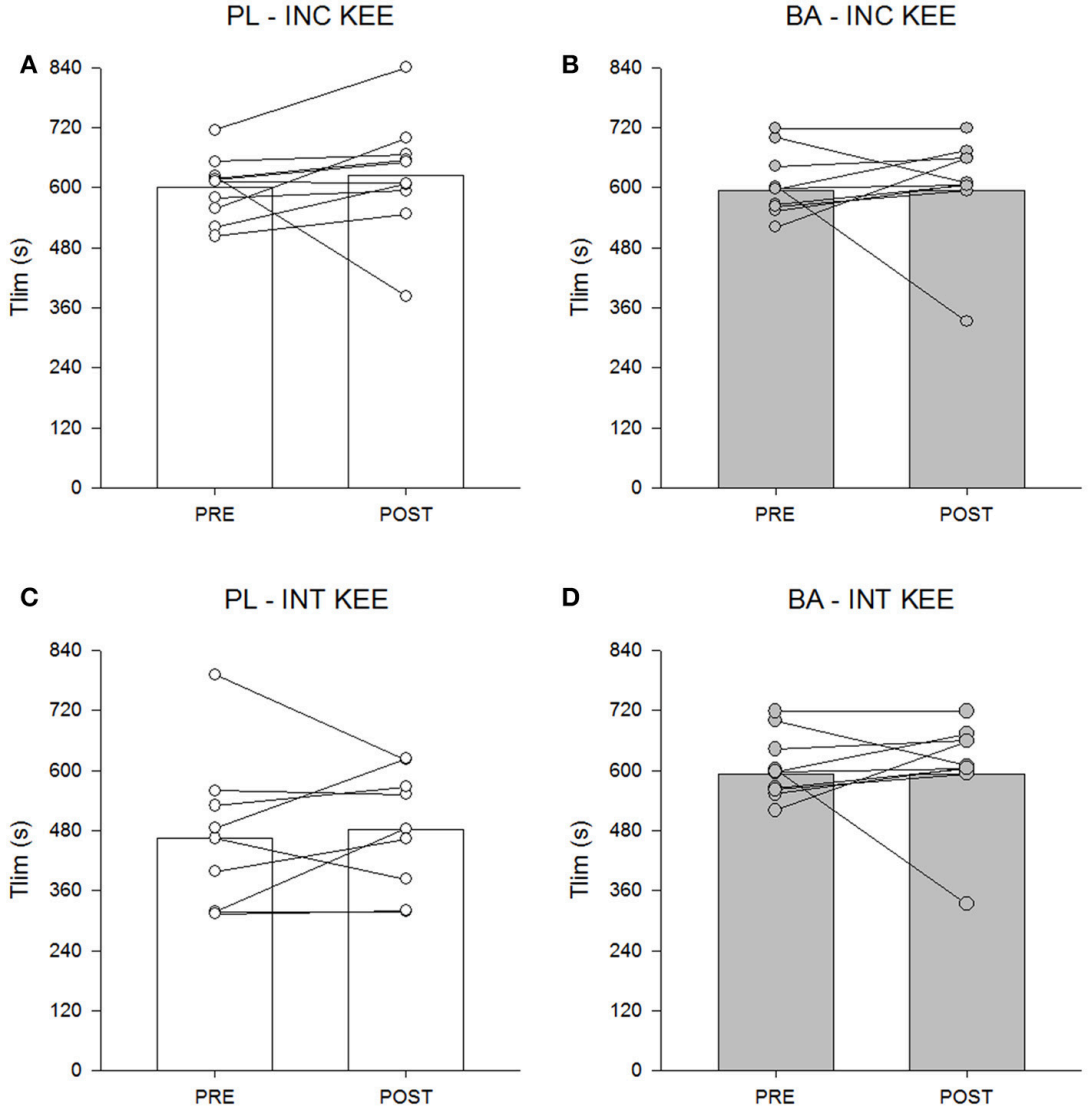
The placebo (PL; white) and β-alanine (BA; gray) group mean and individual T_lim_ during incremental (INC KEE) **(A,B)** and intermittent (INT KEE) **(C,D)** knee-extension exercise pre- (circles) and post- (triangles) supplementation.

There was no significant between group difference for INC KEE or INT KEE performance following supplementation (*P* > 0.05) (Figure 6).

## Discussion

We employed a comprehensive exercise testing regimen, which included whole-body and single-legged exercise modalities and the use of ^1^H- and ^31^P-magnetic resonance spectroscopy to determine muscle carnosine content and muscle metabolic changes during exercise, respectively, to investigate the influence of BA supplementation on exercise performance. The principal findings of this study were that BA supplementation did not significantly increase muscle carnosine content or alter intramuscular pH or performance during incremental or intermittent knee-extension exercise, or alter the power-duration relationship during all-out cycling. However, BA supplementation attenuated the fall in blood pH and improved performance by 2 ± 3% during ramp incremental cycle exercise. Although there was great inter-individual variability in muscle carnosine responses to BA supplementation, no relationships were observed between muscle carnosine content and blood pH or exercise performance.

The findings of the current study indicate that muscle carnosine content was not increased following 4 and 6 weeks of BA ingestion (6.4 g·d^−1^). This is in contrast to previous studies that have assessed muscle carnosine content using ^1^H-MRS and have shown that BA supplementation results in increased carnosine content in muscles of the calf (Baguet et al., 2009, 2010; del Favero et al., 2012; Stegen et al., 2013, 2014; Bex et al., 2014; Danaher et al., 2014; Hoffman et al., 2015), thigh (Harris et al., 2006), and upper arm and shoulder (Bex et al., 2014). Whilst these studies employed a variety of different supplementation strategies, and although baseline muscle carnosine content and loading rates appear to be muscle specific (Baguet et al., 2009, 2010; Stegen et al., 2013, 2014; Bex et al., 2014; Danaher et al., 2014), they have consistently reported ~45% increase in muscle carnosine following a total BA dose of ~181 g during the supplementation period. In the present study, subjects had ingested a total of 179 g BA after 4 weeks and 269 g BA after 6 weeks. Given the large increases in muscle carnosine content reported previously, our method should have been sufficiently sensitive (CV ~15%) to detect changes between groups following BA supplementation. A large inter-individual variability was observed in the relative change in muscle carnosine content in response to BA supplementation (−51 to 31%). This finding is similar to Hill et al. (2007) and Baguet et al. (2009) who reported a range of −1 to 161% and 2 to 69%, respectively. Whilst a greater baseline carnosine content has been observed in human type II muscle fibers (Suzuki et al., 2002; Hill et al., 2007), both type I and type II muscle phenotypes have been shown to respond equally well to BA supplementation (Hill et al., 2007; Baguet et al., 2009). Therefore, individual differences in muscle fiber type composition are unlikely to explain inter-individual variation in muscle carnosine response to BA supplementation. The training status of the muscle may influence the magnitude of increase in carnosine, with a greater relative increase reported in more highly exercised muscle groups following BA supplementation, possibly due to a greater delivery of BA to the muscle mediated by an increased blood flow and increased capillary density, and/or a contraction induced stimulation of taurine (TauT) and H^+^/amino acid 1 (PAT1) transporters which facilitate BA uptake into the myocyte (Bex et al., 2014). Given that the subjects in the current study were matched at baseline, were not trained in any particular sport, and that there was no association between muscle carnosine increase and parameters of fitness (i.e., V°O_2peak_, GET, CP, W′), it is unlikely that these factors can explain the inter-individual differences in muscle carnosine responses we observed. It has been shown that L-histidine concentrations decrease by ~27 and ~31% relative to baseline following 12 and 23 days of 6 g·d^−1^ BA supplementation, respectively (Blancquaert et al., 2017). It is possible that reduced L-histidine bioavailability at baseline and as a consequence of BA supplementation may, in part, explain differences in muscle carnosine responses between subjects in the current study and in previous research (Harris et al., 2006; Baguet et al., 2009; Stellingwerff et al., 2012; Stegen et al., 2013, 2014; Bex et al., 2014). A novel finding of our study is that 4–6 weeks of BA supplementation may not always result in a measurable increase in muscle carnosine content (cf. Hill et al., 2007). The factors regulating muscle carnosine content require further research.

The ergogenic effect of BA supplementation has been primarily attributed to its role in the synthesis of muscle carnosine, a potent intramuscular pH buffer (Bate-Smith, 1938). However, to our knowledge there has only been one study that has previously assessed muscle buffering capacity in humans following BA supplementation and, despite observing an increase in muscle carnosine, found no improvements in muscle buffering capacity (Gross et al., 2014). In the current study, we used ^31^P-MRS to assess muscle pH during single-legged knee-extension exercise. It was shown that BA supplementation did not result in changes in muscle pH at rest or during INC KEE or INT KEE and no performance improvement was observed. In addition to INT KEE, we used a repeated 3-min all-out cycling test to determine whether BA supplementation might improve recovery from intense whole-body exercise. The second bout of the 3-min all out test was used to determine the amount of work that could be performed above CP (W > CP) and thus served as an index of W′ recovery. In agreement with Saunders et al. (2012a), but in contrast with Saunders et al. (2012b) who studied the effects of BA supplementation on intermittent exercise, recovery following sprint exercise was not improved in the present study. This observation is consistent with there being no significant change in muscle carnosine content and no change in muscle pH or performance during INT KEE following BA supplementation.

There was a small but possibly meaningful change in blood pH and performance during the ramp incremental cycling test following BA supplementation, despite no significant change in muscle carnosine content. However, it is important to point out that the mean increase of ~2% in ramp test peak power in the BA group is similar in magnitude to coefficients of variation shown for ramp test peak power across 4–6 weeks of placebo supplementation (2.5 %, present study) and 4–5 weeks with no dietary or exercise intervention (1.1%; Lindsay et al., 1996). The increased blood pH and ramp test performance are in contrast with previous studies that have shown no significant improvements in incremental test performance following BA supplementation (Zoeller et al., 2007; Van Thienen et al., 2009), and the present finding that BA supplementation did not alter the power-duration relationship or blood pH during the 3-min all-out test. The CP and W′, when estimated using a conventional protocol of multiple prediction trials, have also been shown to be unaffected by BA supplementation (Smith-Ryan et al., 2012). The 3-min all-out test has been shown to be sensitive to detect changes in the power-duration relationship following training (Vanhatalo et al., 2008b) and acute normobaric hypoxia (Simpson et al., 2015), but no effects on CP or W′ were found following sodium bicarbonate ingestion, despite an elevated blood pH (Vanhatalo et al., 2010). Therefore, dietary interventions that may transiently enhance muscle (present study; Smith-Ryan et al., 2012) or blood (Vanhatalo et al., 2010) buffering capacity in some individuals do not appear to result in significant changes to the power-duration relationship.

The effects of BA supplementation on high-intensity exercise performance are equivocal (meta-analysis see Hobson et al., 2012; Saunders et al., 2017), with improved exercise performance having been reported by some (e.g., Saunders et al., 2012b; Hoffman et al., 2015) but not all (e.g., Sweeney et al., 2010; Saunders et al., 2012a; Ducker et al., 2013; Jagim et al., 2013) previous studies. The discrepancy between findings does not appear consistently linked to differences in supplementation regimes or exercise test protocols. Ducker et al. (2013) and Jagim et al. (2013) observed no effect of BA supplementation despite following the same supplementation regime (6.0 g·d^−1^ for 4 weeks) as Hoffman et al. (2015). The repeated performance of short sprint-intervals (Sweeney et al., 2010; Saunders et al., 2012a; Ducker et al., 2013) or high-intensity constant work-rate protocols (Jagim et al., 2013), which result in task failure in ~1–2.5 min, would be expected to require substantial contributions from glycolysis and thus to decrease muscle pH (Bogdanis et al., 1995, 1998; Vanhatalo et al., 2016). Test duration and supplementation strategy is therefore unlikely to explain the lack of differences observed during the knee-extension protocols, the power-duration relationship, and W > CP in the current study. Although interpretation of the studies reporting no significant improvements following BA supplementation is limited due to the omission of a carnosine assessment (Sweeney et al., 2010; Saunders et al., 2012a; Ducker et al., 2013; Jagim et al., 2013), it seems reasonable to suggest that BA supplementation may not have sufficiently increased muscle carnosine content in most individuals within these studies.

### Experimental considerations

It was not possible to assess muscle carnosine content on every laboratory visit due to the large number of tests. It is possible that a temporal lag of 3–4 days between some performance test visits and muscle carnosine scans influenced the accuracy of correlations between muscle carnosine and exercise performance indices. Previous research has shown, however, that muscle carnosine content is relative stable and has a slow wash-out rate following BA supplementation (Stellingwerff et al., 2012). There was no significant difference in muscle carnosine between 4 and 6 weeks of BA supplementation in the present study, although some individual variability was evident (Figure 1). It may be speculated that muscle carnosine content was elevated in the majority of the subjects in the BA group at the time of the ramp incremental test but not at the time of the 3-min all-out tests. This seems unlikely, however, and the randomization of exercise tests would have minimized any consistent order effect. It should be considered that there are substantial differences in the dynamics of muscle activation, glycolytic rate, muscle H^+^ efflux, blood flow and the rate of W′ utilization between the ramp incremental and 3-min all-out sprint test protocols. These differences might have contributed to the small performance benefit observed in the ramp test but not the all-out sprint test.

We assessed muscle carnosine in a 1.5 T scanner by quantifying the integral of the carnosine peak relative to the integral of the water peak, which enabled assessment of relative changes in muscle carnosine from pre- to post-supplementation. It should be noted, that all previous studies using ^1^H-MRS to assess muscle carnosine content have used a 3.0 T scanner, which would improve the signal-to-noise ratio. In the present study, the CV % between baseline scans was similar when performed on consecutive days (15 ± 8%) compared to scans separated by 2 weeks (i.e., week 4 and 6 of supplementation; 18 ± 16%), 4 weeks (15 ± 13%), and 6 weeks (18 ± 16%). We can therefore be confident that: (1) the technique we used would have been sufficiently sensitive to detect the changes in muscle carnosine that have been reported previously (Harris et al., 2006; Baguet et al., 2009, 2010; del Favero et al., 2012; Stegen et al., 2013, 2014; Bex et al., 2014; Danaher et al., 2014; Hoffman et al., 2015); and (2) we did not observe an increase greater than the measurement error. In keeping with the lack of change observed in muscle carnosine content and thus buffering capacity, we found no differences in muscle pH during INC and INT KEE. Collectively, these findings strongly suggest that the supplementation regime did not successfully increase muscle carnosine content and muscle buffering capacity. Given that adherence to the supplementation regime was confirmed by each subject, it should also be considered that the supplement did not contain the expected dosage of BA. The possible absence of active ingredients in some commercially-available dietary supplements has been noted as a concern previously (Maughan, 2013). However, given that the supplementation product used in this study had been tested to ensure that it contains the identity and quantity of ingredients indicated on the label (NSF Certified for Sport), supplement contamination, and decreased presence or omission of the active ingredient seems unlikely. Why we did not observe a significant increase in muscle carnosine and thus muscle buffering capacity having used a certified supplement, followed an adequate BA loading strategy, and utilized a sufficiently sensitive method for carnosine detection, is unclear.

## Conclusions

A variety of high-intensity exercise tests comprising different work-rate forcing functions and exercise modalities were used to assess possible ergogenic effects of BA supplementation. Under the conditions of the present study, BA supplementation had a variable and non-significant effect on muscle carnosine content and no influence on intramuscular pH during high-intensity incremental or intermittent knee-extension exercise. The small increase in blood pH during ramp incremental cycle exercise following BA supplementation was associated with a small but significantly greater increase in performance relative to the PL group but this was not sufficient to alter the power-duration relationship. Our findings indicate that BA supplementation may not always increase muscle carnosine content, and clearly, in such circumstances, no effect on exercise performance would be expected.

## Ethics statement

This study was carried out in accordance with the recommendations of University of Exeter Research Ethics Committee with written informed consent from all subjects. All subjects gave written informed consent in accordance with the Declaration of Helsinki. The protocol was approved by the University of Exeter Research Ethics Committee.

## Author contributions

MB, AJ, and AV were involved in conceptual design, data collection, interpretation of results, and manuscript preparation; PM, JF, and SB were involved in data collection, interpretation of results, and manuscript preparation. MB, AJ, AV, PM, JF, and SB approved the final version of the manuscript and agreed to be accountable for all aspects of the work.

### Conflict of interest statement

The authors declare that the research was conducted in the absence of any commercial or financial relationships that could be construed as a potential conflict of interest.
